# Post-implementation Review of the Himalaya Home Care Project for Home Isolated COVID-19 Patients in Nepal

**DOI:** 10.3389/fpubh.2022.891611

**Published:** 2022-05-17

**Authors:** Rakchya Amatya, Kritika Mishra, Kshitij Karki, Isha Puri, Archita Gautam, Sweta Thapa, Urmila Katwal, Siddhesh Veer, John Zervos, Linda Kaljee, Tyler Prentiss, Kate Zenlea, Gina Maki, Pawan Jung Rayamajhi, Narendra K. Khanal, Pomawati Thapa, Madan Kumar Upadhyaya, Deepak Bajracharya

**Affiliations:** ^1^GTA Foundation, Lalitpur, Nepal; ^2^Global Health Initiative, Henry Ford Health, Detroit, MI, United States; ^3^Henry Ford Hospital, Department of Infectious Disease, Detroit, MI, United States; ^4^Curative Service Division, Department of Health Services, Ministry of Health and Population, Kathmandu, Nepal; ^5^Quality Standard and Regulation Division, Ministry of Health and Population, Kathmandu, Nepal

**Keywords:** COVID-19, Himalaya Home Care (HHC), home isolation, counseling activities, telehealth (TH), Nepal

## Abstract

**Background:**

The emergence of coronavirus disease 2019 (COVID-19) has resulted in a pandemic that has significantly impacted healthcare systems at a global level. Health care facilities in Nepal, as in other low- and middle-income countries, have limited resources for the treatment and management of COVID-19 patients. Only critical cases are admitted to the hospital resulting in most patients in home isolation.

**Methods:**

Himalaya Home Care (HHC) was initiated to monitor and provide counseling to home isolated COVID-19 patients for disease prevention, control, and treatment. Counselors included one physician and four nurses. Lists of patients were obtained from district and municipal health facilities. HHC counselors called patients to provide basic counseling services. A follow-up check-in phone call was conducted 10 days later. During this second call, patients were asked about their perceptions of the HHC program. Project objects were: (1) To support treatment of home isolated persons with mild to moderate COVID-19, decrease burden of hospitalizations, and decrease risks for disease transmission; and, (2) To improve the health status of marginalized, remote, and vulnerable populations in Nepal during the COVID-19 pandemic.

**Results:**

Data from 5823 and 3988 patients from May 2021-February 2022 were entered in initial and follow-up forms on a REDCap database. The majority of patients who received counseling were satisfied. At follow-up, 98.4% of respondents reported that HHC prevented hospitalization, 76.5% reported they could manage their symptoms at home, and 69.5% reported that counseling helped to limit the spread of COVID-19 in their household.

**Conclusions:**

Telehealth can be an essential strategy for providing services while keeping patients and health providers safe during the COVID-19 pandemic.

## Introduction

On March 11, 2020, the World Health Organization (WHO) declared COVID-19 a global pandemic (1, 2). COVID-19 causes respiratory illness resembling two previous outbreaks - severe acute respiratory syndrome coronavirus [SARS-CoV] and Middle East respiratory syndrome coronavirus [MERS-CoV] (3). The clinical spectrum and severity of COVID-19 ranges from mild to severe life-threatening symptoms (4, 5). Individuals with serious symptoms are advised to seek immediate medical attention, while those with mild symptoms are advised to manage their symptoms at home (1). The first COVID-19 case in Nepal was detected on January 23, 2020 (6). Delta and Omicron genetic variants contributed to surges in cases in April-June 2021 and January 2022, respectively (7, 8). Through March 1, 2022, there were over 977,000 identified cases, among which 90.5% have recovered and 1.3% have died. Among the total active cases, 85% were in home isolation (9).

Prior to the pandemic, there was a scarcity of physicians, nurses and paramedics in Nepal. Additionally, health care facilities had limited resources for the treatment and management of COVID-19 (10–13). With a sizeable number of patients in home isolation, the Nepali health system was challenged to reach and counsel these patients in disease treatment and prevention. To address this need, GTA foundation (GTA) in coordination with Department of Health Service (DoHS) under the Ministry of Health and Population (MoHP) and the Henry Ford Health Global Health Initiative (HFHS GHI) initiated the Himalaya Home Care (HHC) helpline. The implementation of HHC was adapted from a successful helpline call center quality improvement project piloted by the Henry Ford Health. The HFHS project was deployed to address the surge of COVID-19 cases and hospitalizations in Detroit as a rapid response and community engagement effort. Data collection on patient outcomes was obtained.

The focus of HHC was to determine the feasibility and outcomes of a similar model in a low- or middle-income setting, for future scale-up and more rigorous outcome measurements. The GHI team collaborated with HFHS COVID Command Center to obtain the scripting for patient counseling, diagnosis, and treatment guidelines, FAQs for COVID-19 exposure, and the variables used for data collection.

## Materials and Methods

### Program Description

The HHC team consisted of a multi-disciplinary team, including a physician, nurses, data analysts and IT support staff. HHC was implemented between May 2021 and February 2022. The HHC operated 12 h/day, 7 days per week. The call center was housed at the National Public Health Laboratory in Kathmandu. The project focused on services for rural and remote areas of Nepal and was available in all seven provinces in Nepal. Districts were selected in collaboration with the MoHP with regards to low-resource settings, including rural areas with limited access to healthcare providers in the district. The basic counseling services provided to patients included: (1) a check on patient's health status (symptoms) and prescribing treatment/medications for those symptoms; (2) information about signs and symptoms indicative of possible increase in disease severity; (3) principles of home isolation; (4) prevention measures to decrease spread of disease in the household; (5) mental health information and prioritizing need for relaxed schedules and maintaining communication with friends and family; (6) information on possible post-COVID effects; and, (7) awareness regarding the importance of vaccination.

### Program Objectives

Primary - To support treatment of home isolated persons with mild to moderate COVID-19, decrease burden of hospitalizations, and decrease risks for disease transmission.

Secondary - To improve the health status of marginalized, remote, and vulnerable populations in Nepal during the COVID-19 pandemic.

### Participant Enrollment

**Figure 1:**
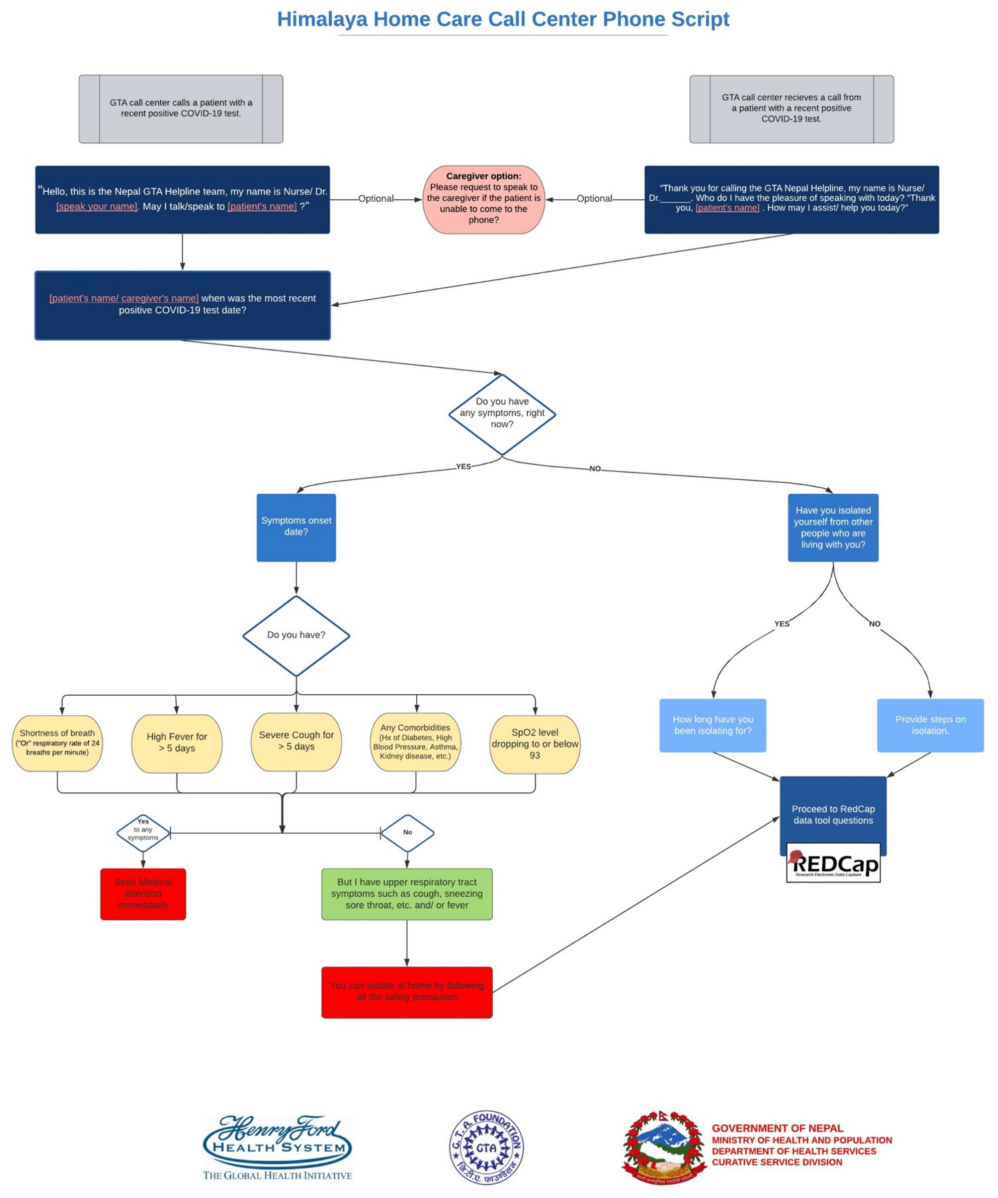
Himalaya home care call center phone script.

HHC received lists of COVID-19 home isolated patients from district and municipal health facilities on a weekly basis. The lists were received in password protected pdf and excel files via emails. A total of 17,563 patients were identified. Identified patients included persons who were home isolated, hospitalized, those who had died from COVID-19, and who tested positive twice for the same case of COVID-19. Only home isolated patients were contacted. The counselors completed 13,335 initial calls and 5,754 follow-up calls (43.1%). Data were recorded from 5,823 patients during the initial calls and 3,988 patients during follow-up (68.5%) (Figure 1). Counselors reported that the average call times were between 5 and 7 min. Follow-up calls were conducted 10 days after the initial call. The difference in initial and follow-up calls was due to the death of patients, admission to hospitals or isolation centers, and difficulty in contacting patients.

### Data Collection, Management, and Analysis

The HHC counselors collected and managed patient data using REDCap (Research Electronic Data Capture) hosted at GTA Nepal (14, 15). REDCap is a secure, web-based software platform designed to support data capture. Initial and follow-up call data were linked through a unique record ID. The following data was collected during the initial call:

*Demographic data:* Respondent Gender (M/F/Binary/No Response), Age.

*Household data:* Province, Number of people in household, Number of COVID-19 positive people in the household at time of the call, Availability of a separate room for isolation in the home (Y/N).

*Vaccination status:* Receipt of at least one dose of a COVID-19 vaccine (Y/N).

*Consultations:* Management of COVID-19 (Y/N), Referral for diagnostic testing (Y/N), Hospital referral (Y/N), Information on best practices during home isolation (Y/N).

During the follow-up calls, data were collected on respondents' satisfaction with the HHC program. These items included: (1) HHC supported prevention of hospitalization (Y/N); (2) HHC supported symptoms management at home (very helpful/somewhat helpful/not helpful); and (3) HHC supported prevention of spread of COVID-19 within the household (very helpful/somewhat helpful/not helpful). The GHI team also created a dashboard to report daily updates on patient enrollment, patient satisfaction and vaccination status data to our partners in Nepal and Detroit.

For the data analysis, frequencies and percentages were reported for categorical variables. Mean was reported for continuous variables. Chi-square test, *t*-test and ANOVA test were used in bivariate analysis to measure associations between independent variables (gender, age, province, vaccination status) and dependent variable (data collection time points, vaccination status and patient satisfaction). A *p*-value of <0.05 was considered statistically significant.

## Results

### Demographic and Household Characteristics

Most of the patients who received counseling through initial and follow-up calls were male (55.6 and 55.1%, respectively). Mean age at the initial call and follow-up was 36.6 and 36.7 years. There was no significant difference between the initial and follow-up call by demographics.

The average number of people living in households was 4.8 and the average number of COVID-19 positive people in households was 2.1. Over three-fourth of the patients (77.5%) had a separate room for isolation purposes. Over half of the counseled patients were from Province 1 (54.1%). Karnali (2.1%) and Sudurpashchim (2.6%) provinces had the least number of participants.

### Vaccination Status

There was a significant difference by gender, age and province in terms of COVID-19 vaccination status (received at least one dose). More male respondents and older respondents had received at least one dose of vaccine compared to female and younger respondents. Most of the respondents were not vaccinated. By province, the highest rate of reported vaccination was in Lumbini and the lowest in Karnali (Table 1).

**Table 1:** Respondent vaccination status (at least one dose) by gender, age, and province.

**Item**	**Category**	**Response % (** ***N*** **)**		***p*-value**
		**Vaccinated (Yes)**	**Vaccinated (No)**	
Gender	Female	33.6% (644)	66.4% (1,270)	0.021
	Male	37.1% (846)	62.9% (1,436)	
Age (Mean/SD)		43.8 (SD 16.3)	33.9 (SD 15.4)	<0.001
Province	Province 1	33.6% (838)	66.4% (1,655)	<0.001
	Madhesh Province	33.7% (85)	66.3% (167)	
	Bagmati Province	35.5% (215)	64.5% (391)	
	Gandaki Province	37.3% (118)	62.7% (198)	
	Lumbini Province	48.5% (147)	51.5% (156)	
	Karnali Province	36.2% (42)	63.8% (74)	
	Sudurpashchim Province	40.9% (45)	59.1% (65)	

### Counseling Services and Referrals

HHC provided consultation on disease management, referral for diagnostic testing, hospital referral for treatment and provision of information on home isolation/ quarantine during initial calls. The HHC staff counseled over 99% patients on managing COVID-19 symptoms at their home and on best practices for home isolation/quarantine. Less than one percent of the counseled patient were referred for further diagnostic testing [0.7% (40)] and/or referred to the hospital for treatment based on their symptoms [0.5% (26)].

### Participants' Perception of the HHC Program

During the follow-up call, the counselor asked the respondents about their satisfaction with the HHC program in terms of (1) prevention of hospitalization; (2) symptom management at home; and (3) prevention of disease spread within the household.

### Prevention of Hospitalization

Among the total patients counseled, a majority felt that the HHC program prevented hospitalization. Younger patients were more likely to report that the program prevented hospitalization compared to older patients. Over 98.0% of the patients from all the provinces were prevented from hospitalization regardless of their vaccination status (Table 2).

**Table 2:** “Prevention of hospitalization” by gender, age, province, and vaccination status.

**Item**	**Category**	**Response % (** ***N*** **)**		***p*-value**
		**Yes**	**No**	
Gender	Female	98.0% (1,450)	2.0% (29)	0.109
	Male	98.8% (1,592)	1.2% (20)	
Age (Mean/SD)		36.5 (SD 17.2)	42.2 (SD 16.2)	0.021
Province	Province 1	98.2% (1,929)	1.8% (36)	0.403
	Madhesh Province	98.4% (63)	1.6% (1)	
	Bagmati Province	99.5% (394)	0.5% (2)	
	Gandaki Province	98.1% (262)	1.9% (5)	
	Lumbini Province	98.4% (241)	1.6% (4)	
	Karnali Province	98.3% (59)	1.7% (1)	
	Sudurpashchim Province	100.0% (94)	0	
Vaccination Status	Yes	98.5% (1,076)	1.5% (16)	0.698
	No	98.3% (1,666)	1.7% (28)	

### Symptom Management

An equal proportion of male and female patients thought that the counseling related to management of COVID-19 symptoms at home was very helpful and somewhat helpful. There was no difference by gender or age in relation to perceptions of this portion of the program. A majority of the respondents in all the provinces felt the counseling was “very helpful” for symptom management. However, there was a significant difference in perceptions by province (*p* < 0.05). Vaccinated respondents were also more likely to report that the program was effective in symptom management compared to those who had not received a vaccine dose (*p* < 0.05) (Table 3).

**Table 3:** “Symptom management” and “Preventing spread of COVID-19” by gender, age, province, and vaccination status.

**Item**	**Category**	**Response % (** ***N*** **)**			***p*-value**
		**Very helpful**	**Somewhat helpful**	**Not helpful**	
**Symptom management**					
Gender	Female	76.5% (1,131)	23.1% (341)	0.5% (7)	0.946
	Male	76.5% (1,233)	23.0% (370)	0.6% (9)	
Age		36.9(17.0)	35.6 (17.5)	39.4(16.4)	0.142
Province	Province 1	76.6% (1,506)	23.1% (453)	0.3% (6)	<0.001
	Madhesh Province	82.8% (53)	17.2% (11)	0	
	Bagmati Province	83.8% (332)	14.4% (57)	1.8% (7)	
	Gandaki Province	74.5% (199)	25.1% (67)	0.4% (1)	
	Lumbini Province	66.1% (162)	33.1% (81)	0.8% (2)	
	Karnali Province	78.3% (47)	21.7% (13)	0	
	Sudurpashchim Province	69.1% (65)	30.9% (29)	0	
Vaccination status	Yes	82.8% (904)	17.0% (186)	0.2% (2)	<0.001
	No	73.4% (1,243)	25.9% (439)	0.7% (12)	
**Preventing spread of COVID-19**					
Gender	Female	68.9 (973)	29.5 (417)	1.6 (23)	0.092
	Male	70.1 (1,089)	29.2 (453)	0.8 (12)	
Age		36.9 (16.8)	36.4 (17.8)	33.6 (18.8)	0.403
Province	Province 1	69.8 (1,313)	29.1 (547)	1.1 (21)	0.008
	Madhesh Province	84.1 (53)	15.9 (10)	0.0 (0)	
	Bagmati Province	73.7 (272)	23.8 (88)	2.4 (9)	
	Gandaki Province	66.2 (174)	33.1 (87)	0.8 (2)	
	Lumbini Province	62.0 (147)	37.1 (88)	0.8 (2)	
	Karnali Province	71.7 (43)	28.3 (17)	0.0 (0)	
	Sudurpashchim Province	63.8 (60)	35.1 (33)	1.1 (1)	
Vaccination status	Yes	76.3 (803)	23.4 (246)	0.3 (3)	<0.001
	No	66.0 (1,068)	32.6 (528)	1.4 (23)	

### Prevention of the Spread of COVID-19 Within Households

Over two-thirds of respondents thought that the counseling was very helpful for prevention of COVID-19 within their households. There was no significant difference in perceptions by gender or age. However, there were differences by province (*p* = 0.008) and vaccination status (<0.001). Those respondents vaccinated were more likely to report that the program was ‘very helpful’ in terms of COVID-19 spread compared to unvaccinated respondents (Table 3).

## Discussion

The Himalaya Home Care (HHC) program provided counseling services to 5,823 home-isolated COVID-19 patients. Implementation of similar programs during the COVID-19 pandemic have also been shown to be effective. COVID-19 related telehealth services have been implemented in many other countries in Asia and globally (16–23). In Bangladesh (22), counseling services were provided through phone calls similar to the HHC program. In India and Bangladesh, psychological counseling was provided to prevent and treat mental health issues arising and exacerbated due to the pandemic (22, 23). In China, live video conferencing was used and the recipient of the counseling services included both clinicians and patients (19). In Malaysia, participants reported that not having to travel to a health center for consultation was the most important element in a telehealth program (16). While the details of the telehealth programs differ, overall, this approach has effectively provided general care and COVID-19 related services to persons and households isolated due to quarantines, shutdowns, and personal concerns (e.g., fear of infection).

In the current study, satisfaction with the program varied somewhat by residency (province) and vaccination status. In terms of the latter, those vaccinated may have had milder symptoms than those not vaccinated and/or have more trust in the government and health system (24). However, overall, there was high satisfaction with the HHC program amongst the patient population in terms of prevention of hospitalization and disease spread within households and symptom management. The focus on populations living in more remote and rural areas provided an opportunity for health counseling and education regarding COVID-19 that otherwise may not have been readily available.

Through HHC, healthcare providers could counsel and support patients from 60 districts within all seven provinces of Nepal. The HHC program included a multidisciplinary team and partnerships between governmental and non-governmental organizations in Nepal and between Nepal and a U.S. health system. These partnerships and the support of the Nepali government were an essential component to the success of the program.

### Study Limitations

The major limitation of this pilot project was the lack of an intervention-control model to determine whether HHC improved outcomes of COVID-positive patients. The project was designed primarily as a feasibility study to understand whether this model was effective, practical, and acceptable for use in a low- and middle-income country. Future iterations of this project will more rigorously study the effect that this or a similar telemedicine services could have on patient outcomes. Another challenge faced by the HHC program was the deployment of a Short Message Service (SMS) outreach communication tool. The purpose of this approach was to provide participants with up-to-date relevant information about COVID-19 and safety guidelines. The tool was not implemented due to technical difficulties with integrating an automated texting service with Nepali telecom services. Other SMS options will be explored to implement with future telemedicine programs in Nepal.

## Conclusion

HHC successfully provided counseling to home isolated COVID-19 patients throughout Nepal. The majority of the patients who received counseling were satisfied with the services. The outcomes from this program indicate that telehealth is a feasible and effective tool for reaching vulnerable and isolated populations in Nepal both during the pandemic and in the future.

## Data Availability Statement

The raw data supporting the conclusions of this article will be made available by the authors, without undue reservation.

## Ethics Statement

Ethical review and approval was not required for the study on human participants in accordance with the local legislation and institutional requirements. Written informed consent for participation was not required for this study in accordance with the national legislation and the institutional requirements. Data was collected as part of a quality improvement program and therefore ethical approval was not required. All patients were consented verbally prior to receiving counseling.

## Author Contributions

RA involved in creating and managing of data collection tool in REDCap, monitoring the project, analyzing the data and drafting the manuscript. KM involved in providing counseling services along with data collection, data entry from COVID-19 patients, and drafting and reviewing the manuscript. KK and DB involved in conceptualization, management of the project, editing, and reviewing the manuscript. IP, AG, ST, and UK involved in providing counseling services along with data collection, data entry from COVID-19 patients, and review of the manuscript. SV involved with creating the data collection tool, the script for the nursing staff conducting patient counselling, HHC dashboard for tracking daily patient enrollment, counseling calls, and COVID care kits distribution data, editing and reviewing the manuscript. Initiated the deployment of an SMS outreach communication tool. JZ, KZ, and TP involved in conceptualization and supported the development of the protocol. LK provided expert comments and guided the team to identify and analyze data points and tables. Helped in editing and reviewing the manuscript. GM reviewed and provided feedback on the selection of data collection variables for the project. PR, NK, PT, and MU- involved in conceptualization, coordination, and communication for the operation of the project, reviewing the manuscript. All authors contributed to the article and approved the submitted version.

## Funding

The project was funded by Henry Ford Health Global Health Initiative, USA.

## Conflict of Interest

The authors declare that the research was conducted in the absence of any commercial or financial relationships that could be construed as a potential conflict of interest.

## Publisher's Note

All claims expressed in this article are solely those of the authors and do not necessarily represent those of their affiliated organizations, or those of the publisher, the editors and the reviewers. Any product that may be evaluated in this article, or claim that may be made by its manufacturer, is not guaranteed or endorsed by the publisher.
